# The impact of lockdowns during the COVID-19 pandemic on work-related accidents in Austria in 2020

**DOI:** 10.1007/s00508-022-02013-2

**Published:** 2022-04-12

**Authors:** Dominikus Huber, Roland Frank, Richard Crevenna

**Affiliations:** 1 Division of Oncology, Department of Medicine I, Medical University of Vienna, Waehringer Guertel 18–20, 1090 Vienna, Austria; 2 Allgemeine Unfallversicherungsanstalt Österreich AUVA, Vienna, Austria; 3 Department of Physical Medicine, Rehabilitation and Occupational Medicine, Medical University of Vienna, Waehringer Guertel 18–20, 1090 Vienna, Austria

**Keywords:** SarsCov2, Occupational injuries, Accidents, Stay at home orders, Teleworking, Social control

## Abstract

**Background:**

This study aims to investigate the impact of the lockdowns during the COVID-19 (Corona-Virus-Disease 19) pandemic in Austria on work-related accidents in the year 2020. Apart from the lockdowns, multiple work-related measures were introduced in 2020, such as the new law on short-term work and regulation on accidents during home-office. Their combined effects on work-related accidents are unknown and a secondary parameter of this study.

**Methods:**

Daily data on the number of accepted and rejected cases of work-related accidents from the Allgemeine Unfallversicherungsanstalt were obtained for the years 2019 and 2020. Based on data provided by the World Health Organization and government publications, the beginning and end dates of national hard and soft lockdown periods were derived. From this database, a difference-in-differences regression analysis on the absolute number of daily work-related accidents was conducted.

**Results:**

On average 272.3 work-related accidents per day were registered in 2019 and 199.4 in 2020, a statistically significant reduction of 72.9 accidents per day and total decrease of 26,164 less accidents compared to 2019. Both lockdowns had a statistically highly significant effect on work-related accidents: The hard lockdown reduced the average number of daily registered work-related accidents by 40%. The light lockdown phases reduced this number by an average of 51%. Weekends and holidays had the greatest impact on work-related accidents with a reduction of 69% and 73%, respectively.

**Conclusion:**

Both lockdown qualities during the COVID-19 pandemic in Austria led to a significant reduction in work-related accidents for their duration. These findings merit further investigation with more detailed data on sectors and injury-quality.

**Supplementary Information:**

The online version of this article (10.1007/s00508-022-02013-2) contains supplementary material, which is available to authorized users.

## Background

Austria provides an interesting case in studying the impact of lockdowns in 2020 on public life, in particular work-related accidents due to a few unique features.

**Fig. 1:**
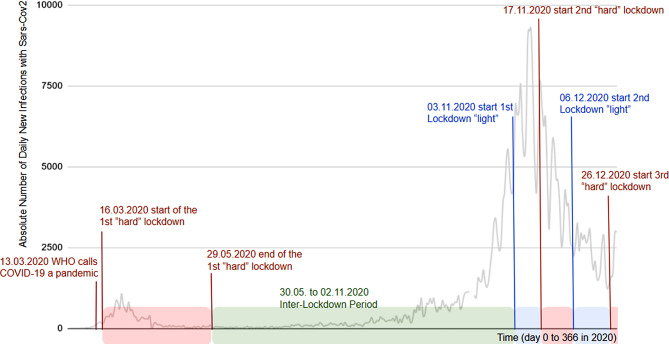
Daily new infections with COVID-19 (Corona-Virus-Disease 19) in Austria as reported by the Federal Interior Ministry and documented by Dong et al. (2020) [9]. Superimposed are in *red* the hard lockdown periods, in *blue* the lockdown light phases and in *green* the inter-lockdown period with close to no restrictions work-wise

At first glance, restrictions to social contacts, both private and at work, were similar to other developed countries in terms of timing and scope. Between 29th of February and 22nd of March most European [1] and Asian countries [2] had implemented decisive restrictions of movement, social interaction and gatherings. In Austria, such policies came into place on Monday, March 16th, 5 days after the WHO declared COVID-19 (Corona-Virus-Disease 19) a global pandemic and 3 days after Europe was declared the epicenter of this pandemic [3, 4]. The first cases in Austria, however, were detected between 24th and 26th of January 2020 [5]. The last of the initial restrictions was lifted in Austria on May 29th [6]. The following lockdowns in the 4th quarter (Q4) of 2020 had two different characters. Between 3rd and 16th of November 2020 and between 6th and 25th of December 2020 social distancing policies similar to the first lockdown period were implemented, with the exception of retail and services staying open [7]. These periods were colloquially called “lock down light” as opposed to the stricter measures of the earlier “hard lockdown”. In reaction to and anticipation of surging cases, another two hard lockdowns were imposed between and after the lock down light phases [8]. This is where Austria is different from other European countries, where lockdown measures remained fairly homogeneous and constant over a longer period of time. This provides the opportunity to study a situation similar to a repeated experiment regarding different lockdown measures in Austria (Fig. 1).

Due to these lockdown restrictions, approximately one third of the working population in Austria and other European countries switched in Q1 2020 to working from home [10]. For this form of teleworking the term “home office” was used in German-speaking countries. Home office was, of course, not possible in all industries and some sectors had to reduce operations significantly. Consequently, production declined in all European countries on average 3.1% and in Austria about 2.8% in Q1 2020 [11]. In Q1 and Q3 2020 people worked about 8% less hours compared to the same quarters from 2019 while in the Q2 the decrease in hours worked was 19% and in Q4 11% [12]. This difference in decline seems to coincide with lockdown phases but data are not available detailed enough to draw conclusions. It is clear, however, that Austrians worked less in total and more often from home during the pandemic, similar to their European neighbors. Newly introduced was a Home Office Bill alongside measures to curb the economic outfall of the lockdown measures in March 2020. The new law extended the definition of work-related accident to all private rooms, including the living room and the kitchen. Moreover, working time was defined more flexibly and the range of covered activities broadened to all activities necessary to satisfy essential needs, including cooking [13]. The combined effect of the increase in telework alongside a broader definition of work accidents at home on the one hand and reduced hours worked and reduced production on the other has not been studied before.

Work accidents in Austria are defined as all sudden events that influence health from outside the body and that are related to the activity that is insured under the § 175 Abs (1) ASVG (Arbeits und Sozialversicherungsgesetz, “Work and Social-Security Law”) [14]. This quite unique formulation is a product of a historically grown distinction between traumatologic or toxicologic workplace accidents and chronic, internal diseases that may arise from an occupation. As a result, the data set used in this study, which was derived from Allgemeine Unfallversicherungsanstalt (AUVA, General Accident Insurance Institution), consists of only patients with acute traumatological needs. This is important, since most European countries, including Austria, accepted a COVID-19 infection as work-related disease in their insurance catalogue. These cases are, however, not included in the AUVA database. The following analysis of the impact of covid-related measures on work-related accidents only covers classical accidents without the COVID cases.

Several sequential, intensity-modulated lockdown periods, novel legislation on and a surge in working from home workplaces, and a unique way of recording work-related disease make Austria an interesting case in analyzing the effects of lockdowns on numbers of work-related accidents.

## Material and methods

### Data acquisition

The main dataset of work-related accidents on a daily basis from January 1st 2019–December 28th 2020 was obtained from the records of the AUVA. The numbers are the sum of all cases of patients checking into one of the several AUVA hospitals in Austria. Before aggregation on a national level, all cases that were checked into an AUVA hospital but were then determined not to satisfy the criteria of a work-related accident were subtracted from these numbers. The number of daily work-related accidents used in this data set therefore only consists of patients that satisfy the aforementioned definition of such an acute injury at the workplace.

Additional data on gross domestic product, unemployment, and working hours were gathered from Eurostat on a European level and Statistik Austria on national level and cited where used. The start and end dates of the lockdowns were extracted from the respective laws available on the online Law Information System (Rechtsinformationssystem) of the federal government [15].

### Lockdown definition

The aim of this study is to evaluate the impact of the lockdown phases on the number of work-related accidents in Austria in 2020 based on the available data. Here, a year-on-year difference-in-differences regression analysis was chosen to estimate said impact. A dummy was created for the intervention group (the year 2020) with 2019 being used as the control or reference year. The second dummy variable denotes the intervention date. In this study, the proclamation of the global COVID-19 pandemic on March 13th was chosen as the beginning of the intervention period. The interaction term of this variable with the intervention group should capture all pandemic-specific events, such as legislation, altered risk behavior of patients and economic developments. Another two dummy variables account for the two types of lockdown phases: the hard lockdowns from March 16th–May 29th, November 17th–December 5th and December 26th–December 31st and the lockdown light phases from November 3rd–November 16th and December 6th–December 25th. It is important to note here that these days are treated as lockdown phases in both years but are only expected to become significant in the intervention year 2020: The lockdown days in 2019 should not be different from other days in 2019. Only the interaction term of pandemic year 2020 and lockdown dates should produce a significant effect. 

### Data preparation and processing

The model matches each day in 2020 with the corresponding day in 2019. Since the data set contains only data up to 21st of December 2020, both years are restricted to 362 observations. Since 2020 was a leap year, observation counts in 2019 ends on December 22nd in order to achieve a matching amount of observations. This restriction in the database is of entirely random nature. Nevertheless, it has to be discussed how these measures affect the outcome of the analysis.

Data were stored and processed for further analysis in MS Excel (Microsoft Corporation, 2018. *Microsoft Excel*, Available at: https://office.microsoft.com/excel.). Statistical Analysis was carried out in GraphPad Prism (GraphPad Prism version 9.1.0 for Windows, GraphPad Software, San Diego, CA, USA, available at: www.graphpad.com) and Phyton’s statmodel module [16]. Python’s “statmodel” module was used for seasonal decomposition [16].

An alpha of 0.05 was assumed to constitute statistical significance. Where appropriate, confidence intervals are reported, also using alpha = 0.05. In all cases, two-sided testing is performed. *P*-values were adjusted via a Bonferroni-correction for multiple testing.

For the difference-in-differences estimation to be valid, three major assumptions must hold. The two conditions contained in the stable unit treatment value assumption (SUTVA). Looking at the yearly number of work-related accidents in Austria over the past 15 years, numbers have varied only by a few thousand cases year to year [17]. Therefore, the assumption of parallel trends could hold true. The effect of the number of daily new COVID-19 infections in Austria (Fig. 1; [9]) in the model specified earlier proved to be insignificant, suggesting that if this number is taken as a proxy for the pandemic’s intensity, the latter had no significant effect on the experiment. Using two different years (with the last week cut off) as intervention and control group, spill over effects are highly unlikely and therefore, all three SUTVA prerequisites are assumed to be true. Initial data analysis reveals non-normally distributed variable values and a simple poisson model displaying significant overdispersion with a variance-to-mean ratio of over 60. The model was estimated as a negative binomial model with loglink due to the data being counts of events (work-related accidents registered by the AUVA) of the dependent variable.

The final statistical model looks as follows (see also Table 1):

$$ln\_ wra=\beta _{0}+\beta _{1}*D\_ 2020+\beta _{2}*D\_ \textit{pandemic}+\beta _{3}*D\_ 2020*D\_ \textit{pandemic}+\beta _{4}*D\_ hard+\beta _{5}*D\_ \textit{light}+\beta _{6}*D\_ we+\beta _{7}*D\_ \textit{holiday}+ln\_ lag1$$

**Table 1:** Complete list of variables in the final model (*left column*) and their composition (*right column*). Dummy variables are employed as binary variables that assume the value 1 if certain conditions are met and 0 else

Variable	Variable composition
Intercept	Intercept
D_2020	Equals 1 for days in the year 2020, 0 otherwise
D_pandemic	Equals 1 for the days after March 13th each year, 0 otherwise
D_2020*D_pandemic	Equals 1 for the days within the pandemic, 0 otherwise
D_hard	Equals 1 for days falling in hard lockdowns, 0 otherwise
D_light	Equals 1 for days falling in light lockdowns, 0 otherwise
D_we	Equals 1 for weekend days, 0 otherwise
D_holiday	Equals 1 for national holidays, 0 otherwise
lag1_wra	Number of work-related accidents with lag 1
wra	Daily number of work-related accidents

With the logarithm of the number of work-related accidents [ln_wra] as the dependent variable, the effect of the hard and light lockdown periods are estimated with the coefficients of [D_hard], and [D_light], respectively. The variables [D_pandemic] and [D_2020] set up the difference-in-differences model. Weekends and holidays are accounted for by the dummy variables [D_we] and [D_holiday]. [ln_lag1] is the logarithm of the first autocorrelation of the dependent variable [ln_wra].

## Results

**Fig. 2:**
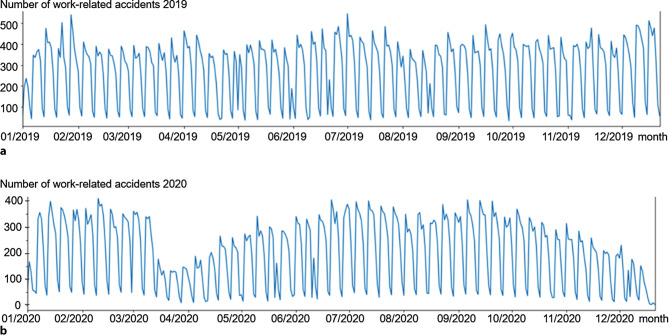
Run-sequence plot of the number of daily work-related accidents registered by all AUVA hospitals in 2019 (**a**) and 2020 (**b**). Both years exhibit a seasonal pattern that is best explained by a drop-in cases on the weekends. The main macroscopically visible differences are sharp decreases in registered cases at the end of March and the beginning of November 2020

The overview over the data in the run-sequence plots on daily work-related accidents in 2020 and 2019 exhibits a similar range, a seasonality with a frequency of about 51 per year and a “dent” in the holiday month August (Fig. 2) during both years. Clearly visible is a decline in numbers of registered accidents in 2020, roughly where one would assume the effects of the lockdowns taking place at the end of Q1 and beginning of Q4.

**Fig. 3:**
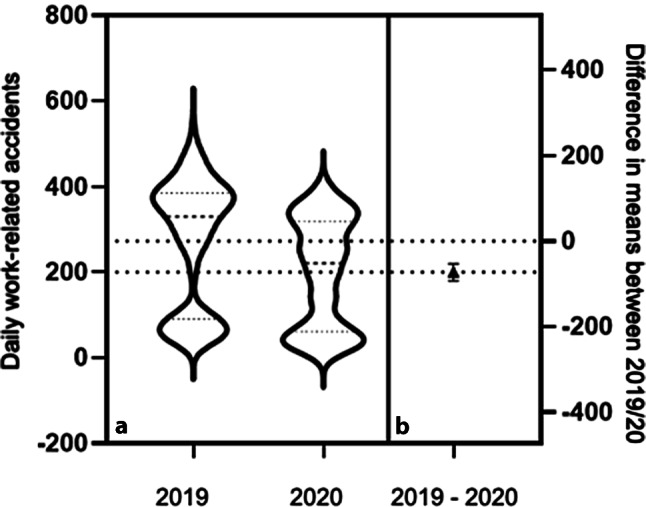
Violin-plot and confidence-interval of the mean difference in work-related accidents 2019 vs. 2020. (**a**) Violin-plot of the daily number of work-related accidents in 2019 (*left violin*) and 2020 (*right violin*). Visualized are the density and distribution of values in both years. The bottom part of both violins looks similar and supports the idea of a seasonal component. The length of the left violin corresponds to a greater range in values and its more pronounced hour-glass shape supports the previous finding of a significant difference in variances between 2019 and 2020. (**b**) Visualization of a highly significant difference in means (*triangle*) with a very narrow 95% confidence interval

In total, the number of registered and accepted work-related accidents in the analyzed time period in 2019 amounted to 97,149 and 70,985 cases in 2020, a minus of 26,164 cases or 26.93%. Clear differences in the data of 2019 and 2020 become visible in the initial statistical analysis (Table 2). In 2019, the daily average of registered work-related accidents amounted to 272.3 cases. In 2020 this number was significantly lower with 199.4 cases. The range of registered cases in 2020 was also significantly lower with 407 cases compared to 511 in 2019. An F‑test on the difference of variances suggests a significantly different distribution of the number of registered cases in both years. These inferential findings are graphically supported by a violin plot (Fig. 3).

**Table 2:** Descriptive statistics of the absolute number of work-related accidents reported in AUVA hospitals in Austria in 2019 and 2020. 2019 had a wider range of values. In 2020, however, an extreme minimum of 3 registered work-related accidents in the entire country on at least 1 day was observed. The mean in both years had a relatively low standard error and therefore rather narrow confidence interval. The F‑test on the difference in variances in the two years (*p* < 0.004) and a t-test on the difference of means (*p* < 0.001) showed highly significant differences

Work-related accidents		
Statistical parameter/year	2019	2020
Minimum	34	3
Maximum	545	410
Range	511	407
Mean	272.3	199.4
Std. deviation	147.4	126.6
Std. error of mean	7.810	6.711
Lower 95% CI of mean	256.9	186.2
Upper 95% CI of mean	287.7	212.6

*Std* Standard Deviation, *CI* Confidence Interval

**Fig. 4:**
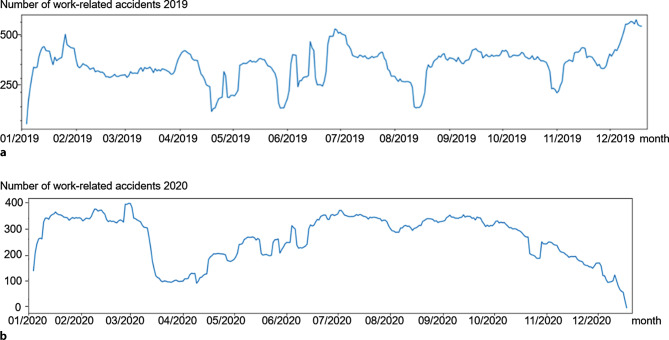
Visualization of the results of a seasonal decomposition of work-related accidents 2019 (**a**) and 2020 (**b**)

A procedure called “seasonal decomposition“ [16] provides a more detailed look at the seasonality of work-related accidents in the years 2019 and 2020. The top panel (year 2019) and bottom panel (year 2020) in Fig. 4 show the final estimate of how work accidents in the respective years would look like if seasonality is removed. After subtracting the weekly component from the original data several outliers in these error values that occur in both years were identified as national holidays in Austria where also a sharp drop in work-related accidents is observed.

A test whether the estimated model showed better fit compared to intercept-only model (“Omnibus Test”) suggested a highly significant improvement to fit of the estimated model (*p* < 0.001). The estimates of the difference-in-differences model are listed in Table 3. Neither the year nor the months corresponding to the pandemic as a whole nor their interaction variable were estimated to have a significant effect on work-related accidents. Both lockdowns, however, had a statistically highly significant effect on work-related accidents. The hard lockdown is estimated to have reduced the average number of daily registered work-related accidents by 39.9%. The light lockdown phases reduced this number by an average of 51.1%. Weekends and holidays were estimated to have the greatest impact on work-related accidents with a reduction of 68.8% and 72.9%, respectively.

**Table 3:** Parameter estimates for the final model of the impact of lockdowns on the number of work-related accidents on a given day. The meaning of each variable name (column 1) is listed in Table 2. Column 2 contains the final estimate of the impact of a given variable as well as that effect’s standard deviation (column 3) and 95% Wald confidence interval (columns 4 and 5). Column 6 gives an impression of the statistical significance of the finding with values below 0.05 being considered statistically significant. In column 7, the effect of each variable on work-related accidents calculated from the estimated coefficient is displayed (e.g. an impact of −0.089 of D_2020 means that compared to 2019, the year 2020 had approximately 9% less work-related accidents)

Variable	B	Standard error	95% Wald confidence interval		Sign.	Impact on wra
			Lower	Upper		
D_2020	−0.093	0.165	−417	−23	0.571	−0.089
D_pandemic	0.046	0.131	−0.211	0.303	0.728	0.047
D_2020*D_pandemic	0.004	0.192	−0.371	0.38	0.981	0.004
D_hard	−0.509	0.141	−0.785	−0.232	<0.001	−0.399
D_light	−0.716	0.198	−1.104	−0.329	<0.001	−0.511
D_holiday	−1.304	0.218	−1.728	−879	<0.001	−0.729
D_weekend	−1.165	0.107	−1.375	−0.955	<0.001	−0.688
ln_lag1	−0.39	0.116	−0.617	−0.164	<0.001	−0.323

*B* estimate of the slope corresponding to a particular variable. *Sign.* *p*-value

## Discussion

Government measures in reaction to the pandemic around the world have raised several questions including economical [18] and ethical [19] discussions alongside the medical debate. Accidents at work touch on all three of these subjects and this study aims to provide a puzzle piece in the discussion with its analysis of the development of work-related accidents during the lockdown periods in Austria. The data analyzed in this study show a significant decline in registered work-related accidents in Austria in 2020 of 26,164 cases (26.93%) compared to the previous year. This drop is very evident when comparing the panels in Fig. 4, where a sharp decline in accident-numbers can be observed shortly after the first lockdown was introduced.

When compared to the available data from other countries, the drop in work-related accidents in Austria in 2020 is immense. Kwon et al. [20] provided an excellent overview on workplace accidents during the pandemic in Korea and elsewhere. They summarized that the number of patients injured at the workplace declined in 2020 by 1664 or 1.8% in Korea and by 2429 cases in Singapore. Japan, on the other side, saw an increase in such cases of 4.4% and in the UK the case count remained fairly stable. Kwon et al. correctly stated that with fewer people going to work, the remaining employees might face tougher working conditions and are more prone to injuries. In most European countries, these numbers are not available until a few years after the year in question. In past years, Austria was with 2.87 injuries per 100,000 persons employed, well above the EU average of 1.77 [21].

However, due to the unique definition of work-related accidents in Austria, an international comparison is difficult. Another possible reason for the discrepancy in the dynamics of work-related accidents during the pandemic could be owed to the difference in lockdown strategies between the aforementioned countries. Korea started already on February 4th with mass testing and implemented a test-and-trace system with the individual isolation of infected patients. No lockdown was implemented in 2020 and the strictest measure was a mandated 2.5 m distance in Q4 2020 [22]. During this time, Austrian authorities were still sceptical about a mass testing strategy and the scientific background inconclusive [23, 24]. Japan, on the other hand, implemented a rather complex COVID-19 response. In accordance with recent legislation, there was no nationwide lockdown in 2020 but only regionally restricted mobility. Moreover, authorities could only request businesses to comply with reduced opening hours and working force but never imposed a mandatory lockdown [25].

It could be argued that government-imposed lockdowns are only valid on paper if not strictly enforced and people still went out, got injured and reported that injury as a “home office” accident, potentially distorting the numbers in this study. Moreover, the lockdowns could have only a delayed effect on people’s behavior, rendering the dummy variables for lockdown phases less significant. A look on Google Mobility Data in 2020 [26] suggests, however, that workplace, transit, and retail mobility measures dropped significantly during the pandemic and that these drops coincided with the implementation dates of the lockdowns (Supplementary Fig. 1). These measures come with several caveats, such as baseline value calculation, selection bias and the fact that many background calculations and definitions by Google remain hidden to the authors of this study. It can therefore only serve as a rough indicator of lockdown effectiveness and work activity.

The effect of lockdowns on the number of registered work-related accidents in Austria estimated by the difference-in-differences model is strong and statistically significant for both lockdown variants. This effect is apparently independent of the pandemic time period from mid-March to December 2020 and all additional measures that were in place during that time but did not change as quickly as the lockdown periods. Such measures could be short-time work (so-called *Kurzarbeit*) or working from home schedules. Interestingly, the light lockdown is estimated to have had a slightly stronger impact than the hard lockdown. This is not explained by the examined data and stands in contrast to the reopening of retail and an increase in travel times for employees compared to the hard lockdown, where no commuting was possible. Further industry sector and country-specific investigation is needed to clarify this finding.

A very clear takeaway is the immense overall reduction in work-related accidents in Austria in 2020 of 26,164 registered accident patients. According to the Austrian Labor Inspectorate and the AUVA, each workplace injury is attributed an average of 12,500 euros of economic costs [27]. In terms of injuries at the workplace, an estimated 327 million euros in economic costs have been saved. This might not seem much compared to other costs the pandemic has caused and what the rehabilitation of COVID patients might still cost [28]. It is a sum, however, that can be used as an incentive to further investigate the lessons from the pandemic work environment.

## Supplementary Information

Supplementary Fig. 1: COVID-19 Google Mobility Report on Austria. Displayed is a % change in movement of users of Google products relative to a baseline [26]. The baseline day is the median value from the 5‑week period Jan 3–Feb 6, 2020 by the company. Clearly visible is a marked drop in all registered mobilities but “residential” on March 16th, the date of the first lockdown in Austria. Similarly, the lockdown series in Q4 2020 entailed a lasting reduction in workplace, retail, and transit mobility.

xxx

## References

[1] Woskie LR, Hennessy J, Espinosa V, Tsai TC, Vispute S, Jacobson BH (2021). Early social distancing policies in Europe, changes in mobility & COVID-19 case trajectories: insights from Spring 2020. PLoS ONE.

[2] Han EMMJT, Turk E, Sridhar D, Leung GM, Shibuya K, Asgari N, Juhwan O, García-Basteiro AL, Hanefeld J, Cook AR, Hsu LY, Yik YT, Heymann D, Clark H, McKee M, Legido-Quigley H (2020). Lessons learnt from easing COVID-19 restrictions: an analysis of countries and regions in Asia Pacific and Europe. Lancet.

[3] Verordnung des Bundesministers für Soziales, Gesundheit, Pflege und Konsumentenschutz gemäß § 2 Z 1 des COVID-19-Maßnahmengesetzes (BGBl. II Nr. 98/2020)

[4] WHO Director-General’s statement on IHR Emergency Committee on Novel Coronavirus (2019-nCoV). 2020. https://www.who.int/director-general/speeches/detail/who-director-general-s-statement-on-ihr-emergency-committee-on-novel-coronavirus-(2019-ncov). Accessed 3 May 2021.

[5] Kreidl P, Schmid D, Maritschnik S (2020). Emergence of coronavirus disease 2019 (COVID-19) in Austria. Wien Klin Wochenschr.

[6] Verordnung des Bundesministers für Soziales, Gesundheit, Pflege und Konsumentenschutz betreffend Lockerungen der Maßnahmen, die zur Bekämpfung der Verbreitung von COVID-19 ergriffen wurden (COVID-19-Lockerungsverordnung – COVID-19-LV) (StF: BGBl. II)

[7] 463. Verordnung des Bundesministers für Soziales, Gesundheit, Pflege und Konsumentenschutz, mit der besondere Schutzmaßnahmen gegen die Verbreitung von COVID-19 getroffen werden (COVID-19-Schutzmaßnahmenverordnung – COVID-19-SchuMaV) available under: https://www.ris.bka.gv.at/Dokumente/BgblAuth/BGBLA_2020_II_463/BGBLA_2020_II_463.pdfsig

[8] 479. Verordnung des Bundesministers für Soziales, Gesundheit, Pflege und Konsumentenschutz, mit der besondere Schutzmaßnahmen zur Verhinderung einer Notsituation auf Grund von COVID-19 getroffen werden (COVID-19-Notmaßnahmenverordnung – COVID-19-NotMV) available under: https://www.ris.bka.gv.at/Dokumente/BgblAuth/BGBLA_2020_II_479/BGBLA_2020_II_479.pdfsig

[9] Dong E, Du H, Gardner L (2020). An interactive web-based dashboard to track COVID-19 in real time. Lancet Infect Dis.

[10] Statistik Austria. Home Office use in Austria in 2020. https://www.statistik.at/web_de/presse/123399.html. Accessed 19 Aug 2021.

[11] Eurostat. NAMQ_10_GDP dataset. https://ec.europa.eu/eurostat/databrowser/bookmark/cc061f42-4c71-414d-a4fc-26aeabf14522?lang=en. Accessed 19 Aug 2021.

[12] Eurostat. NAMQ_10_PE dataset. https://ec.europa.eu/eurostat/databrowser/view/namq_10_pe/default/table?lang=en. Accessed 19 Aug 2021.

[13] Risak M. Arbeitsunfall im Homeoffice: Alles neu durch das 3. COVID-19-Gesetz? CuRe. 2020. https://rdb.manz.at/document/rdb.tso.LIcure20200029.

[14] Allgemeines Sozialversicherungsgesetz § 175, tagesaktuelle Fassung. https://www.ris.bka.gv.at/NormDokument.wxe?Abfrage=Bundesnormen&Gesetzesnummer=10008147&Paragraf=175. Accessed 17 Nov 2021.

[15] Law Information System of the Federal Government in Austria. https://www.ris.bka.gv.at/. Accessed 17 Nov 2021.

[16] McKinney W (2010). Data structures for statistical computing in python. Proceedings of the 9th Python in Science Conference.

[17] Statistik Austria. Mikrozensus-Arbeitskräfteerhebung Ad-hoc-Modul 2013: „Arbeitsunfälle und arbeitsbezogene Gesundheitsprobleme“.

[18] Besley T, Stern N (2020). The economics of Lockdown. Fisc Stud.

[19] Druml C (2020). COVID-19 and ethical preparedness?. Wien Klin Wochenschr.

[20] Baek E-M, Kim W-Y, Kwon Y-J (2021). The impact of COVID-19 pandemic on Workplace accidents in korea. Int J Environ Res Public Health.

[21] Eurostat. Hsw_n2_02 dataset. https://appsso.eurostat.ec.europa.eu/nui/show.do?dataset=hsw_n2_02&lang=en. Accessed 19 Aug 2021.

[22] Normile D (2020). Coronavirus cases have dropped sharply in South Korea. What’s the secret to its success?. Science.

[23] Reiter T, Pajenda S, Wagner L (2021). COVID-19 serology in nephrology healthcare workers. Wien Klin Wochenschr.

[24] Lopes-Júnior LC, Bomfim E, Silveira DSCD, Pessanha RM, Schuab SIPC, Lima RAG (2020). Effectiveness of mass testing for control of COVID-19: a systematic review protocol. BMJ Open.

[25] Osaki T. How far can Japan go to curb the coronavirus outbreak? Not as far as you may think. The Japan Times. Available under https://www.japantimes.co.jp/news/2020/03/01/national/japan-coronavirus-outbreak/#.XlupkCFKiUk. Accessed 19 Aug 2021.

[26] Covid-19 Google Mobility Report. https://datastudio.google.com/reporting/a529e043-e2b9-4e6f-86c6-ec99a5d7b9a4/page/yY2MB?s=ho2bve3abdM. Accessed 19 Aug 2021.

[27] Federal Ministry of Labour, Family, and Youth, Section Labour Law and Labour Security, Austrian Labour Inspectorate. Arbeitsunfälle: Geschichte und Entwicklung der Unfallzahlen. https://www.arbeitsinspektion.gv.at/Uebergreifendes/Arbeitsunfaelle/Arbeitsunfaelle.html. Accessed 19 Aug 2021.

[28] Stam HJ, Stucki G, Bickenbach J, European Academy of Rehabilitation Medicine (2020). Covid-19 and post intensive care syndrome: a call for action. J Rehabil Med.

