# Supplementary material for: The impact of lockdowns during the COVID-19 pandemic on work-related accidents in Austria in 2020
Source: Wien Klin Wochenschr. 2022 Apr 12;134(9-10):391–8. doi: 10.1007/s00508-022-02013-2 (PMC9003159; doi:10.1007/s00508-022-02013-2)
Supplement: Supplementary file 1 — Supplementary Fig. 1: COVID-19 Google Mobility Report on Austria. Displayed is a % change in movement of users of Google products relative to a baseline [26]. The baseline day is the median value from the 5‑week period Jan 3–Feb 6, 2020 by the company. Clearly visible is a marked drop in all registered mobilities but “residential” on March 16th, the date of the first lockdown in Austria. Similarly, the lockdown series in Q4 2020 entailed a lasting reduction in workplace, retail, and transit mobility. [file 508_2022_2013_MOESM1_ESM.docx]

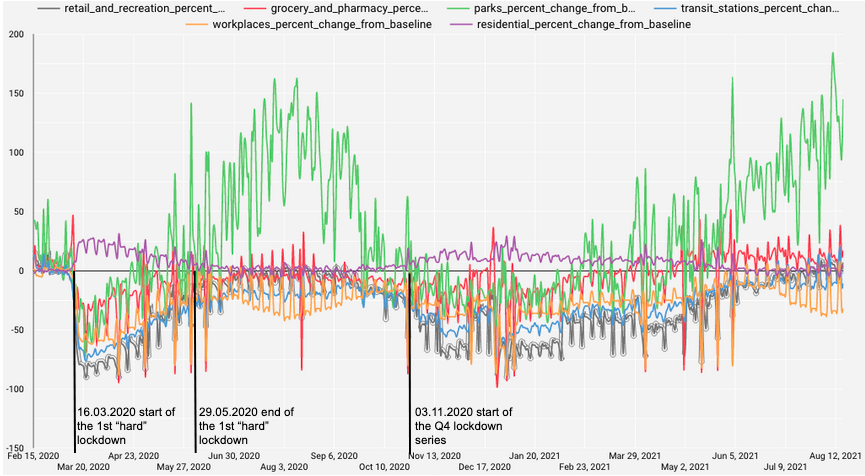


Supplementary Figure 1: COVID-19 Google Mobility Report on Austria. Displayed is a %-change in movement of users of Google products relative to a baseline (28). The baseline day is the median value from the 5‑week period Jan 3 – Feb 6, 2020 by the company. Clearly visible is a marked drop in all registered mobilities but “residential” on March 16th, the date of the first lockdown in Austria. Similarly, the lockdown series in Q4 2020 entailed a lasting reduction in workplace, retail, and transit mobility.
