# Supplementary figures and images for: The impact of lockdowns during the COVID-19 pandemic on work-related accidents in Austria in 2020
Source: Wien Klin Wochenschr. 2022 Apr 12;134(9-10):391–8. doi: 10.1007/s00508-022-02013-2 (PMC9003159; doi:10.1007/s00508-022-02013-2)

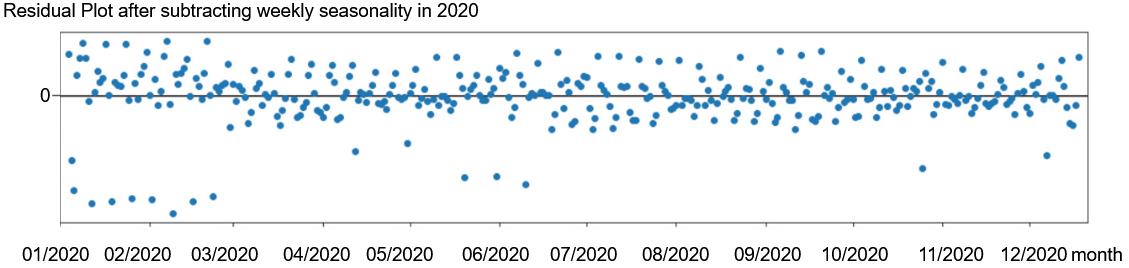


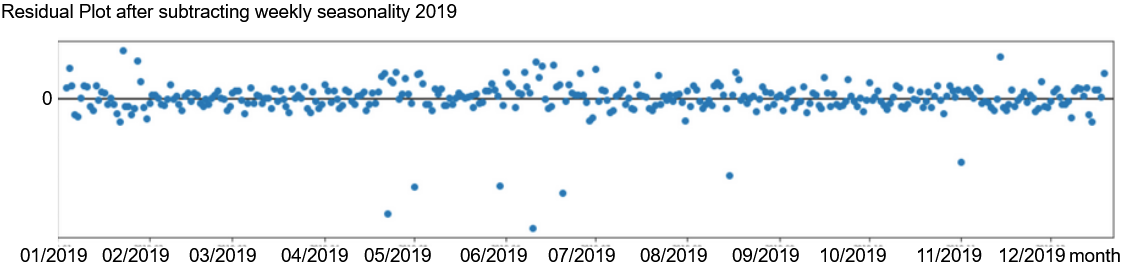

Supplement: Supplementary file 2 — xxx [file 508_2022_2013_MOESM2_ESM.docx]
